# B2, an abscisic acid mimic, improves salinity tolerance in winter wheat seedlings *via* improving activity of antioxidant enzymes

**DOI:** 10.3389/fpls.2022.916287

**Published:** 2022-09-27

**Authors:** Chunxin Yu, Fan Zhou, Ruonan Wang, Zhaojin Ran, Weiming Tan, Linjiang Jiang, Shunyan Cui, Zhouli Xie, Yitao Xiao, Yuyi Zhou, Liusheng Duan

**Affiliations:** ^1^College of Plant Science and Technology, Beijing University of Agriculture, Beijing, China; ^2^State Key Laboratory of Plant Physiology and Biochemistry, College of Agronomy and Biotechnology, China Agricultural University, Beijing, China; ^3^Institute of Biology, Shenyang Research Institute of Chemical Industry Co., Ltd., Shenyang, China

**Keywords:** analogue, salt stress tolerance, antioxidant enzymes, winter wheat seedling, abscisic acid

## Abstract

Salinity severely inhibits growth and reduces yield of salt-sensitive plants like wheat, and this effect can be alleviated by plant growth regulators and phytohormones, among which abscisic acid (ABA) plays a central role in response to various stressful environments. ABA is highly photosensitive to light disruption, which this limits its application. Here, based on pyrabactin (a synthetic ABA agonist), we designed and synthesized a functional analog of ABA and named B2, then evaluated its role in salt resistance using winter wheat seedlings. The phenotypes showed that B2 significantly improved the salt tolerance of winter wheat seedlings by elevating the biomass. The physiological analysis found that B2 treatment reduced the generation rate of O_2_^–^, electrolyte leakage, the content of proline, and the accumulation of malonaldehyde (MDA) and H_2_O_2_ and also significantly increased the contents of endogenous hormones zeatin riboside (ZA) and gibberellic acid (GA). Further biochemical analysis revealed that the activities of various antioxidant enzymes, including superoxide dismutase (SOD), peroxidase (POD), and ascorbate peroxidase (APX), were enhanced by B2, and the activities of antioxidase isozymes SOD3, POD1/2, and APX1/2 were particularly increased, largely resembling ABA treatment. The abiotic stress response-related gene *TaSOS1* was significantly upregulated by B2, while the *TaTIP2;2* gene was suppressed. In conclusion, an ABA analog B2 was capable to enhance salt stress tolerance in winter wheat seedlings by stimulating the antioxidant system, providing a novel regulator for better survival of crops in saline soils and improving crop yield.

## Introduction

Stress caused by excessive soil salt content which inhibits plant growth is called salinity stress (Wilczek et al., 2012). Salt stress is one of the major abiotic stresses that inhibit plant growth and development, and crop production (van Zelm et al., 2020). It is reported that over 800 million ha of land was affected by soil salinization worldwide (Luo et al., 2021). Soil salinization is aggravated by natural environment (e.g., arid climate) and human (e.g., brackish water irrigation) factors, which will threaten food security and bring great economic losses to farmers as well (Munns and Gilliham, 2015). Wheat (*Triticum aestivum* L.) is one of the most important staple food crops worldwide, feeding over 35% of the global population (Li et al., 2022). Due to its salinity sensitivity and soil salinization, the production of wheat displayed a significant loss annually (Garg and Gupta, 1999; Oyiga et al., 2016). Therefore, exploring strategies for improving the salt tolerance of wheat is of great importance for wheat production and sustainability.

The occurrence of salinity stress due to excessive soil salt first causes osmotic stress, which affects water absorption and metabolic response of plants, which is also known as physiological drought (Ibrahimova et al., 2021). High concentrations of Na^+^ and Cl^–^ ions can further disrupt the ionic balance in cells, inhibiting the absorption of other nutrient elements and causing a series of impacts (Gupta and Huang, 2014). Salinity significantly affects plant growth parameters such as plant height, biomass of the shoot and root, leaf area, water content, and seed weight (Goharrizi et al., 2019).

When subjected to salt stress, plants are associated with excessive reactive oxygen species (ROS) synthesis, which causes further oxidative stress. To prevent the damage caused by oxidative stress, plants have developed effective antioxidant systems. Salinity also triggers a variety of physiological and biochemical changes by increasing content of ROS, such as radical superoxide (O_2_^–^), hydrogen peroxide (H_2_O_2_), hydroxyl radicals (OH^–^), and singlet oxygen (^1^O_2_) (Sekmen et al., 2012). Meanwhile, salinity induces a complex antioxidant defense (AOD) system caused by ROS including antioxidant enzymes such as superoxide dismutase (SOD), peroxidase (POD), catalase (CAT) free radical, and enzymes catalyzing ascorbate–glutathione cycle, ascorbate peroxidase (APX), and glutathione reductase (GR) as well as non-enzymatic antioxidants such as carotenoids, glutathione, ascorbic acid, and tocopherols (Pinhero et al., 1997; Lee et al., 2001; Jebara et al., 2005; Zheng et al., 2009; Li et al., 2010). Plants have developed a complex and distinct mechanism to defend salt stress involving morpho-physiological, cellular, and anatomical changes, but the extent to which these mechanisms can improve is limited under severe conditions (Islam et al., 2021). The dynamic balance of ROS plays a role in maintaining this balance (Goharrizi et al., 2019). The exogenous application of different plant growth regulators is a well-recognized strategy to improve crop productivity under stress conditions (Rademacher, 2015). The impact of salt stress on crop growth has been proved to be alleviated by using phytohormones (Javid et al., 2011).

Phytohormones are the critical mediators in response to environmental stresses (Ahmad et al., 2019). Among various phytohormones, abscisic acid (ABA) is one of the most important phytohormones, which provide adaptation to many abiotic stresses, such as salt, drought, and cold stress (Iqbal et al., 2014). Under salt stress conditions, the accumulation amounts of ABA in plants increase, and ABA binds to receptors and activates ABA-responsive pathway to help plants survive. ABA improves salt stress tolerance by stimulating antioxidant defense systems in plants (Jiang and Zhang, 2004). It has been reported that exogenous application of ABA could improve the salt tolerance of sorghum, rice, common bean, etc. (Amzallag et al., 1990; Khadri et al., 2007; Gurmani et al., 2013). However, the application of ABA to enhance crop stress tolerance is largely restricted by its chemical instability, high production cost, and rapid catabolism in plants (Todoroki et al., 2009). A breakthrough discovery identified a chemical on ABA receptors, namely, pyrabactin, which functions like ABA in the aspect of inhibiting seed germination (Park et al., 2009). As it is more structurally stable than ABA and has a low production cost, pyrabactin could be used as a lead structure to design a new family of ABA analogs (Park et al., 2009).

In this study, we designed and synthesized two ABA mimics and tested their effects on the salinity resistance of winter wheat, by investigating biomass, ROS accumulation, and the antioxidant defense system exposed to salt stress. We mainly aim to clarify the protective role of exogenous ABA functional analogs in the antioxidant defense system of winter wheat under salt stress.

## Materials and methods

### Plant materials and growth conditions

The winter wheat (*Triticum aestivum* L.) Jimai 22 was grown in hydroponic conditions in a climate chamber. The seeds were surface-sterilized with 0.5% sodium hypochlorite solution for 15 min, thoroughly washed with distilled water, and germinated in silica sand in a plastic box (50 cm × 30 cm × 10 cm) covered by a black plastic sheet to retain 100% moisture. At the stage of the second leaf emergence, the seedlings with a similar root length were transplanted to the cultivating box with full-strength modified Hoagland nutrient solution under 12/12 h photoperiod, at 25/22°C day/night temperature, 50% relative humidity, and 400 μmol m^–2^ s^–1^ light intensity, and the growth solution was renewed every 3 days. The components of nutrient solution were as follows: 30.0 μM H_3_BO_3_, 2.5 μM ZnSO_4_7H_2_O, 0.8 μM CuSO_4_5H_2_O, 5.0 μM MnSO_4_H_2_O, 100 μM NaFeEDTA, 0.03 μM (NH_4_)_6_Mo_7_O_24_4H_2_O, 2.5 mM Ca(NO_3_)_2_4H_2_O, 0.5 mM NH_4_H_2_PO_4_, 1.0 mM MgSO_4_7H_2_O, and 2.5 mM KNO_3_ (Hoagland and Arnon, 1950; Shen et al., 2009).

### Synthesis and selection of abscisic acid analogs

#### 2,4-Dichlorobenzoyl chloride

A 100 mL three-necked round bottom flask equipped with a mechanical stirrer, addition funnel, and a thermometer was charged with 2,4-dichlorobenzoic acid (1.91 g, 0.01 mol), thionyl chloride (1.19 g, 0.01 mol), and methylbenzene (20 mL). The reaction mixture was refluxed at 80°C for 6 h. The reaction was detected by thin-layer chromatography (TLC) until it is complete. The excess dichlorosulfoxide and toluene were removed by vacuum distillation to obtain liquid aromatic chloride with no further operation.

#### 1-[2-(2,4-Dichlorophenyl)-2-oxoethyl]cyclopropane-1-carboxylic acid (B2)

1-Amino-1-cyclopropanecarboxylic acid (1.0 g, 0.01 mol), sodium hydroxide (0.8 g, 0.02 mol), and deionized water (30 mL) were added into a 100 mL three-necked round-bottom flask equipped with a mechanical stirrer, addition funnel, and a thermometer. The aromatic chloride (2.09 g, 0.01 mol) was added dropwise to the stirred solution. Then, the reaction mixture was stirred at room temperature for 24 h. The reaction was completely detected by TLC. The pH was adjusted to around three by progressively adding concentrated hydrochloric acid, and solid was precipitated out. The mixture was filtered through a filter paper into a solid. The crude product was recrystallized from ethanol and dried, and the target compound was obtained which was a white solid. The yield was 72.3%. Melting point (m.p.): 243–246°C. ^1^H NMR (300 MHz, DMSO) δ 1.05–1.10 (m, 2H, CH_2_), 1.37–1.42 (m, 2H, CH_2_), 7.42–7.52 (m, 2H, benzene-H), 7.67 (s, 1H, benzene-H), 8.99 (s, 1H, NH), 12.47 (s, 1H, COOH). IR (KBr) 3,671, 3,247, 1,708, 1,651. HRMS-ESI m/z calculated for C_11_H_10_Cl_2_NO_3_ [M + H]^+^ 274.00323, found 274.00305.

#### 1-[2-(4-Chlorophenyl)-2-oxoethyl]cyclopropane-1-carboxylic acid (B5)

1-[2-(4-Chlorophenyl)-2-oxoethyl] cyclopropane-1-carboxylic acid (B5) was synthesized from 1-amino-1-cyclopropanecarboxylic acid (1.0 g, 0.01 mol), sodium hydroxide (0.8 g, 0.02 mol), and 4-chlorobenzoyl chloride (1.75 g, 0.01 mol) and purified by recrystallization from ethanol. The target compound was obtained as a white solid. The yield was 59.6%. Melting point (m.p.): 218-220°C. 1H NMR (300 MHz, DMSO) δ 1.08–1.12 (m, 2H, CH_2_), 1.38–1.43 (m, 2H, CH_2_), 7.84 7.59 (m, 4H, benzene H), 9.05 (s, 1H, NH), 12.35 (s, 1H, COOH). IR (KBr) 3,671, 3,265, 1,708, 1,641. HRMS-ESI m/z calculated for C_11_H_11_ClNO_3_ [M + H]^+^ 240.04220, found 240.04193.

**FIGURE 1:**
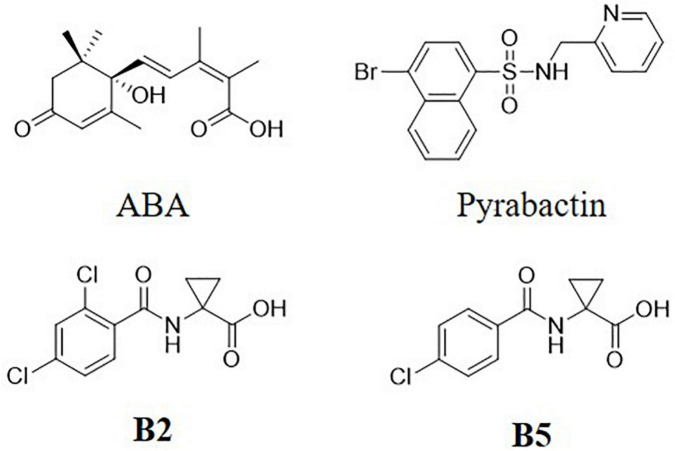
Structures of abscisic acid (ABA) and the analogs.

Structures of B2 and B5 were showed in Figure 1. The two compounds were screened by the inhibitory effect on seed germination activity (Zhou et al., 2013; Han et al., 2015; Supplementary Table 1).

### Treatments and experimental design

The seedlings were pretreated by culturing in Hoagland nutrient solution containing ABA, B2, or B5 at a concentration of 0.01 μM for 24 h and then were cultured under normal (Hoagland nutrient solution) or salinity conditions (Hoagland nutrient solution with 150 mM NaCl). The experimental design was followed as given Table 1. Each treatment was independently replicated three times and arranged in a completely randomized design.

**TABLE 1:** Treatments of experimental design.

Treatments	Nutrient solution	150 mM NaCl	0.01 μM ABA	0.01 μM B2	0.01 μM B5
Control (CK)	√	–	–	–	–
ABA (A)	√	–	√	–	–
B2 (B)	√	–	–	√	–
B5 (B5)	√	–	–	–	√
Salt (S)	√	√	–	–	–
Salt + ABA (S + A)	√	√	√	–	–
Salt + B2 (S + B)	√	√	–	√	–
Salt + B5 (S + B5)	√	√	–	–	√

After 6 days of salinity treatment, the youngest fully expanded leaf of each treatment was sampled immediately in liquid nitrogen and stored at –80^°^C for the determination of free oxygen radicals, malonaldehyde (MDA) level, solute leakage, 1,1-diphenyl-2-picrylhydrazyl (DPPH) radical scavenging activity, and antioxidant enzymes.

The seedlings were treated for 11 days and then dried at 80°C for 24 h (Azri et al., 2020). The seedling dry weights of both root and shoot were measured individually. The root/shoot ratio was calculated as the root dry weight divided by the corresponding shoot dry weight.

### Determination of reactive oxygen species generation

The ROS generation was expressed as the accumulation of O_2_^–^ and H_2_O_2_. The content of O_2_^–^ was determined by monitoring the formation rate of nitrite from hydroxylamine according to Verma and Mishra (2005). The assay was performed by homogenizing 0.3 g frozen leaves in 4 mL 50 mM phosphate buffer (pH 7.8) in an ice bath and then centrifuging at 12,000 r/min for 15 min at 4°C. The supernatant was then subject to quantification of O_2_^–^. The formation rate of O_2_^–^ was calculated from the standard curve of NaNO_2_ reagent.

H_2_O_2_ was extracted following the method of Okuda et al. (1991). A measure of 0.3 g frozen leaf was homogenized in 200 mM ice-cold perchloric acid. After centrifugation at 3,000 r/min for 10 min, the supernatant was transferred into a new tube and neutralized with 4 M KOH. Insoluble potassium perchlorate was eliminated by centrifugation at 3,000 r/min for 10 min. The reaction was started by the addition of peroxidase (horseradish, J&K Scientific Co., Ltd., Beijing, China), and the increase in absorbance was recorded at 590 nm for 3 min (UV-6000PC, Shanghai Metash Instruments Co., Ltd., Beijing, China).

### Assay of lipid peroxidation and membrane permeability

Lipid peroxidation in the leaf was determined by measuring the amount of MDA using thiobarbituric acid (Dhindsa et al., 1981). A measure of 0.3 g sample was homogenized with 4 mL 10% trichloroacetic acid and centrifuged at 8,000 r/min for 15 min at 4°C. The supernatant was mixed with 0.6% thiobarbituric acid at a 1:1 ratio and then incubated at 100°C for 15 min. The absorbance of the reaction solution was determined at wavelengths of 450, 532, and 600 nm.

Membrane permeability was determined by relative conductivity (L_*t*_/L_0_) as described in Lutts et al. (1996). The latest fully expanded leaves from three seedlings were washed three times with distilled water to remove surface-adhered electrolytes and were then cut into 1 cm segments per treatment. The tissue segments were placed in stoppered vials with 10 mL distilled water and were incubated in the dark at 25°C in a rotary shaker at 120 r/m. After 24 h, the electrical conductivity of the bath solution (L_*t*_) was measured. The samples were then autoclaved for 30 min to achieve 100% electrolyte leakage, and a final conductivity reading (L_0_) was recorded upon equilibration at 25°C.

### Estimation of soluble sugar and soluble protein contents

The soluble sugar content in the leaf was evaluated at 620 nm using a spectrophotometer following the anthrone method (Fales, 1951). Fresh leaves (0.5 g) were put into 15 ml of distilled water and boiled in a water bath for 20 min. After cooling, 5 mL of anthrone was added to 0.1 mL of the boiled sample. Then, 3 ml of the boiled sample was transferred to a cuvette, and the absorbance was read. Finally, the content of total soluble sugar in the samples was calculated using a standard glucose curve.

The soluble protein concentration was estimated following the method described by Sedmak and Grossberg (1977) in a microplate spectrometer (Multiskan FC, Thermo Fisher Scientific Inc., United States). A standard curve of bovine serum albumin was used to determine soluble protein concentrations.

The proline content was determined following the method of Koca et al. (2007). The leaf samples (0.3 g) from each treatment were homogenized in 5 mL of 3% (w/v) sulfosalicylic acid and then soaked in a boiling water bath for 10 min. After cooling, 1 mL of supernatant was boiled in a 100°C water bath for 1 h after the addition of 2 mL of 2.5% acid ninhydrin and 1 mL of glacial acetic acid. The mixture was placed in an ice bath to stop the reaction and extracted with 4 mL of toluene. After static stratification, the toluene phase was taken to determine the absorption value at 520 nm. Proline concentration was calculated using a calibration curve.

### Determination of 1,1-diphenyl-2-picrylhydrazyl radical scavenging activity

The DPPH radical scavenging activity was determined following the method of Sairam and Srivastava (2002). After 0.2 g fresh leaves were homogenized in 3.0 mL pure ethanol at 4°C and centrifuged at 8,000 r/min for 15 min, 0.2 mL supernatant was mixed with 0.5 mL 0.5 mM freshly prepared DPPH, 1.8 mL absolute ethanol, and 2 mL 0.1 M acetate buffer (pH 5.5). The reaction mixtures were shaken vigorously and incubated for 30 min at room temperature. Then, the absorbance of the mixture was measured at 517 nm, and DPPH radical scavenging activity was calculated as described by Jao and Ko (2002).

### Determination of hormone contents

An indirect ELISA technique was used to determine the content of hormones, including ABA, JA, ZR, and gibberellic acid (GA). Extraction and purification of hormones were carried out according to Yang et al. (2001). The specificity of monoclonal antibodies and other possible non-specific immunoreactive interference were checked by Wu et al. (1988), Zhang et al. (1991), and He (1993) and proved reliable.

### Activity analysis of antioxidant enzymes

The enzyme activities of CAT, POD, APX, and GR were measured as described by Parida et al. (2004). Fresh leaves (0.5 g) were ground using a chilled pestle and mortar, and homogenized in 2 mL of sodium phosphate buffer (50 mM, pH 7.0) at 4°C as described by Gossett et al. (1994). The buffer contained 2% polyvinylpyrrolidone, 0.1 mM EDTA, 1 mM D isoascorbic acid, and 0.05% (w/v) Triton X-100. After centrifuging at 10,000 r/min for 30 min at 4°C, the supernatants were collected and used for the detection. Then, one unit of enzyme was taken as the amount of enzyme required to disintegrate 1 μmol of the substrate in 1 min at 25°C.

Catalase activity was measured using the disappearance rate of H_2_O_2_ at 240 nm (Sairam and Srivastava, 2002). The reaction mixture contained 50 mM potassium phosphate (pH 7.0) and 10.5 mM H_2_O_2_ (Miyagawa et al., 2000). Then, the enzyme extract (containing 20 μg of protein) was added to the reaction mixture, and the reaction was run at room temperature for 2 min. The activity was calculated with an absorbance at 240 nm of the initial linear rate of decrease. The activity was calculated with the initial linear rate of decrease in an absorbance at 240 nm.

Guaiacol peroxidase activity was determined spectrophotometrically at 25°C (Tatiana et al., 1999). The reaction mixture contained 50 mmol/L potassium phosphate (pH 7.0), 2 mM H_2_O_2_, and 2.7 mM guaiacol. The enzyme extract (equivalent to 5 μg protein) was added to 2 mL of the reaction mixture to start the reaction. The formation of tetraguaiacol was measured at 470 nm (ε = 26.6 mmol/L^–1^cm^–1^).

The activity of ascorbate peroxidase was measured according to Kuk et al. (2003). The reaction mixture contained 50 mM potassium phosphate (pH 7.0), 0.5 mM ascorbic acid, 0.2 mM EDTA, and 0.25 mM H_2_O_2_. The enzyme extract (containing 50 μg of protein) and H_2_O_2_ were added to the reaction mixture in turn. Then, the reaction was started at 25°C. The absorbance decrease was recorded at 290 nm for 1 min, and the amount of oxidized ascorbic acid was calculated based on the extinction coefficient of 2.8 mmol/L^–1^cm^–1^.

Glutathione reductase was assayed at 25°C by measuring NADPH oxidation rate through the absorbance decrease at 340 nm (ε = 6.2 mmol/L^–1^cm^–1^) following to the method of Klapheck et al. (1990). The reaction mixture (1 mL) was composed of 100 mM Tris–HCl (pH 7.8), 0.005 mM NADPH, 21 mM EDTA, 0.5 mM oxidized glutathione (GSSG), and the enzyme extract. The reaction was started by the addition of NADPH.

For the analysis of superoxide dismutase, the leaf tissue (1 g) was ground with a chilled pestle and mortar, and homogenized in 8 mL of potassium phosphate buffer (50 mM, pH 7.8), which contained 1% insoluble PVP and 0.1 mM Na_2_EDTA. After centrifugation at 20,000 r/min for 20 min, the supernatant was collected and used for the detection of SOD according to the method of Beyer and Fridovich (1986). The reaction mixture contained 27 mL of 50 mM potassium phosphate (pH 7.8), 1.5 mL of 30 mg/mL L-methionine, 1 mL of 1.44 mg/mL nitroblue tetrazolium salt, and 0.75 mL of Triton X-100. The enzyme extract (20 μL) and 44 mg/L riboflavin (10 μL) were added to the reaction mixture (1 mL). Then, the mixtures were illuminated by exposing to light of 20 W fluorescent tubes for 7 min. The increase in absorbance at 560 nm was read, and the activity of SOD was calculated by measuring the percentage inhibition per minute. Overall, 50% of inhibition was defined as equivalent to 1 unit of SOD activity.

### Activity staining of antioxidase isozymes

The activity staining was carried by native polyacrylamide gel electrophoresis (PAGE) following the method of Laemmli (1970). The samples were loaded onto the gels after mixed with 10% glycerol (v/v) and 0.25% bromophenol blue. Protein extract (40 μg) was applied in each lane. The electrophoretic run was completed at a current of 35 mA at 4°C.

Different SOD isoenzymes were separated using 4% stacking gel and 12% running gel at 4°C (Xie et al., 2007). After electrophoresis, the gels were soaked in 50 mM phosphoric acid buffer (pH = 7.8), containing 0.03 mM riboflavin, 0.25 mM NBT, and 28 mM TEMED for 30 min in the dark at room temperature. Then, the gels were irradiated with a fluorescent lamp to start the photochemical reaction, and SOD was localized.

Different CAT isoenzymes were separated on 7.5% polyacrylamide gel containing 0.5% soluble starch at 4°C following the method of Kuk et al. (2003). After electrophoresis, the gels were immediately incubated in a solution for 30 s at 25°C, which contained 18 mM sodium thiosulphate and 679 mM H_2_O_2_. Then, the gel was successively washed with distilled water and 90 mM potassium iodide solution containing 0.5% glacial acetic acid. Negative bands of CAT isoenzymes appeared on the gel with a blue background.

Different POD isoenzymes were separated using 4% stacking gel and 5.5% running gel at 4°C (Parida et al., 2004). The gels were dyed in the staining solution [containing 50 mM phosphoric acid buffer (pH = 5.0), 0.03 mM riboflavin, 0.1 mM EDTA-Na_2_, 0.25 mM NBT, and 28 mM TEMED] for 30 min in the dark.

Different APX isozymes were separated on 7.5% non-denaturing polyacrylamide gel (containing 1.1 M sorbitol and 1 mM Na-ascorbate) at 4°C (Parida et al., 2004). After electrophoresis, the gels were stained in 50 ml of 100 mM potassium phosphate buffer (pH 6.4) containing 20 mM guaiacol and 5.55 mM H_2_O_2_. After washing two times with 10 mM potassium phosphate (pH 6.0), the gels were incubated in the same buffer containing 4 mM Na ascorbate and 4 mM H_2_O_2_ for 15 min at 25°C. The gels were rinsed with water and incubated with oscillations for 3 min. The gels were then immersed in the solution of 2.4 mM potassium ferricyanide.

Activity staining of GR was performed on 7.5% PAGE following the method of Rao et al. (1996). After electrophoresis, the gels were rinsed with distilled water and immersed in staining solution (50 mL of 25 mM Tris–HCl buffer, pH 7.5, containing 10 mg MTT, 3.4 mM GSSG, 10 mg DCPIP, and 0.5 mM NADPH) until GR isozyme bands appeared on the gel.

### RNA extraction and quantitative reverse transcription polymerase chain reaction assay

Reverse transcription polymerase chain reaction (RT-qPCR) analysis and leaf tissues of different treatments were used for total RNA extraction. Total RNA extraction and quantitative polymerase chain reaction (qPCR) assay were conducted, as described by Zheng et al. (2018). β*-Actin* was used as an internal control. Primer sequences for qPCR were designed according to the coding sequence of *TaSOS1* and *TaTIP2;2* by using Primer 5 software and checked using the BLAST search in the apple genomic database. The primer sequences are shown in Supplementary Table 2. For each sample, three individual repeats of biological experiments were used for statistical analysis.

### Statistical analysis

A completely randomized design was adopted with three biological replicates for physiology in this study. The experiments were carried out in triplicate. Data were analyzed using ANOVA in SAS (SAS Institute, Cary, NC, United States). *P* < 0.05 was considered statistically significant (Student’s *t*-tests).

## Results

### Effect of exogenous treatments on plant biomass

**FIGURE 2:**
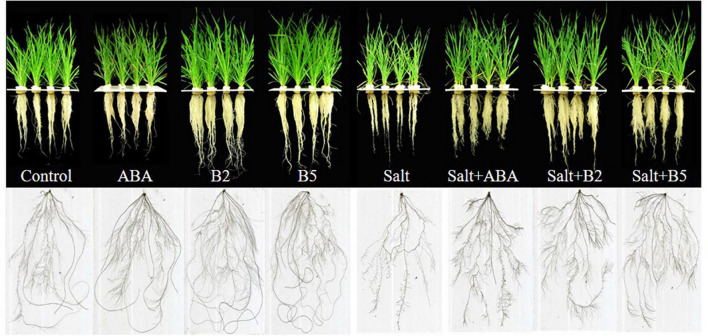
Morphology of wheat seedlings with different exogenous treatments under normal and salinity conditions. Wheat seedlings at three-leaf stage were incubated in Hoagland solution with a particular chemical for 24 h and were then transferred into fresh Hoagland solution with or without 150 mM NaCl. Pictures were taken at 11 days after salt treatment. CK, control; ABA, ABA treatment under normal conditions; B2, B2 treatment under normal conditions; B5, B5 treatment under normal conditions; Salt, salinity treatment; salt + ABA, ABA treatment under salt stress; salt + B2, B2 treatment under salt stress; salt + B5, B5 treatment under salt stress.

To characterize the effects of ABA and ABA analogs on salt tolerance in wheat seedling growth, we measured the biomass of wheat seedlings under different treatments. Different responses to salinity were observed in control and pretreated seedlings (Figure 2). After salt treatment for 11 days, leaf yellowing was more severe in untreated seedlings than in the pretreated seedlings. Salinity stress also altered root elongation and reduced the growth rate of wheat seedlings, which lead to the reduction of biomass. Under both normal and salinity conditions, wheat seedlings treated with different small molecules did not show significant differences in biomass, including shoot dry weight, root dry weight, total dry weight, root/shoot ratio, and plant height of wheat seedlings (Table 2).

**TABLE 2:** Shoot dry weight, root dry weight, total dry weight, root/shoot ratio, and plant height of wheat seedlings treated with B2 and B5 after salt treatment for 11 days.

Treatment	Shoot dry weight (g/plant)	Root dry weight (g/plant)	Total dry weight (g/plant)	Root/Shoot ratio	Plant height (cm)
Control	0.151 ± 0.028 cde	0.051 ± 0.010 cd	0.20 e	0.348 ± 0.017 d	35.1 ± 1.441 cd
ABA	0.179 ± 0.036 abc	0.066 ± 0.011 ab	0.25 bc	0.353 ± 0.032 d	33.7 ± 1.222 de
B2	0.203 ± 0.023 a	0.073 ± 0.007 a	0.27 a	0.363 ± 0.045 cd	39.8 ± 0.513 a
B5	0.188 ± 0.017 ab	0.069 ± 0.016 ab	0.26 b	0.393 ± 0.051 bcd	37.7 ± 1.466 b
Salt	0.064 ± 0.013 f	0.023 ± 0.002 e	0.08 f	0.337 ± 0.011 d	27.6 ± 1.909 f
Salt + ABA	0.145 ± 0.028 de	0.056 ± 0.007 bcd	0.21 e	0.390 ± 0.037 b	32.8 ± 1.120 e
Salt + B2	0.161 ± 0.007 bcd	0.069 ± 0.012 ab	0.23 d	0.427 ± 0.062 abc	33.4 ± 1.602 de
Salt + B5	0.130 ± 0.023 e	0.063 ± 0.013 abc	0.19 e	0.480 ± 0.038 a	33.1 ± 0.680 de

Means (*n* = 5) followed by the same letter are not significantly different from each other within the same column at *P* = 0.05.

Under non-stressed conditions, the total dry weight was significantly increased by 25%, 35%, and 30%, respectively, by the application of ABA, B2, and B5 compared with the control. The plant height was significantly increased by 13.4 and 7.4% by the application of B2 and B5 compared with the control, but not significant in ABA treatment. Under salt-stressed conditions, the wheat seedlings were seriously damaged in salt treatment, and the total dry weight of seedlings was reduced by 60% compared with the control. The exogenous application of ABA, B2, and B5 remarkably increased total dry biomass to 260, 290, and 240%, respectively, compared with the plants under salt treatment and alleviated the effect of salt stress on wheat seedlings. Moreover, a greater root/shoot ratio was observed in pretreated wheat under the salt-stressed condition, indicating that exogenous application increased root growth more than shoot growth. Surprisingly, the biomass of B2-treated plants under salt-stressed conditions was significantly higher (15%) than that of the control plants under normal conditions. The results indicated that B2 enhanced plant growth more than ABA and B5 and could even enhance the biomass of wheat while eradicating the growth restriction posed by salt stress.

### Effects of exogenous B2 on the reactive oxygen species generation and cell membrane

**FIGURE 3:**
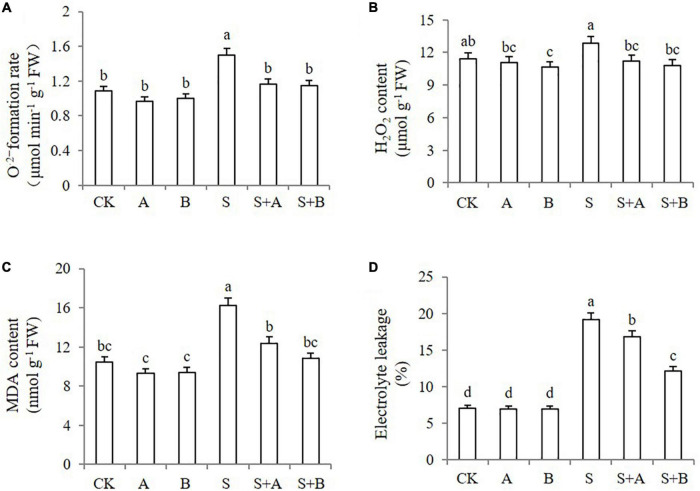
Effects of B2 on ROS generation, peroxidation, and membrane permeability in wheat seedlings after chemical treatments for 6 days. **(A)** Formation rate of O_2_^–^. **(B)** H_2_O_2_ content. **(C)** MDA content. **(D)** Electrolyte leakage. CK, control; A, ABA treatment under normal conditions; B, B2 treatment under normal conditions; S, salinity treatment; S + A, ABA treatment under salt stress; S + B, B2 treatment under salt stress. Data are presented as treatment mean with SE (*n* = 3). Bars with the same letter are not significantly different at *P* = 0.05.

As the accumulation of ROS in plants can be induced by salt stress, we next investigated ROS levels in the leaves under different treatments by monitoring the accumulation of O_2_^–^ and H_2_O_2_. Under normal conditions, the generation rate of O_2_^–^ in young wheat leaves was not affected by the applications of ABA and B2 (Figure 3A). As expected, the plants exhibited increased accumulation of both O_2_^–^ and H_2_O_2_ upon salt stress. The generation rate of O_2_^–^ was sharply induced by salinity stress but was reduced by 22.67 and 23.33%, respectively, when exogenous ABA and B2 were applied. Salt stress significantly increased the content of H_2_O_2_, which was reduced by the application of ABA and B2 under salt-stressed conditions (Figure 3B). The reduction was also observed under B2 treatment under normal conditions.

To determine whether the increased ROS level induced cell injury, we evaluated the MDA content and electrolyte leakage. The plants without pretreatment exhibited an increased accumulation of MDA and electrolyte leakage under salt conditions compared with those under normal conditions (Figure 3C). ABA and B2 could reduce the MDA content by 23.6 and 33.0%, respectively, under salt-stressed conditions, compared with untreated conditions. B2 also decreased electrolyte leakage by 36.5% after salt treatment (Figure 3D). Lower levels of MDA and electrolyte leakage indicated that the cell membrane integrity was protected by ABA or B2, due to their reduction on ROS.

### Effects of exogenous B2 on the osmolytes

**FIGURE 4:**
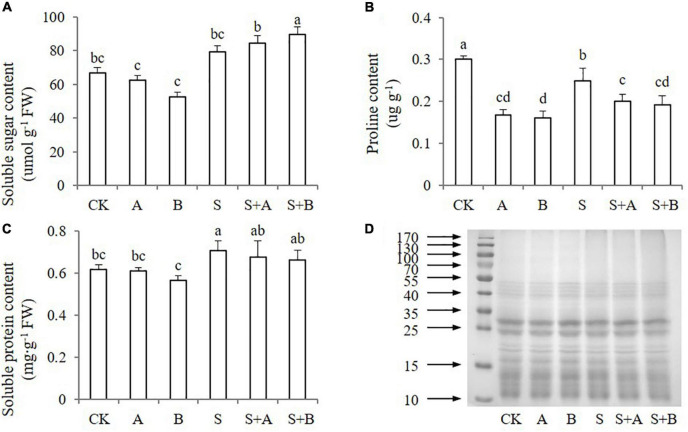
Effects of B2 on osmolytes in wheat seedlings after salt treatments for 6 days. **(A)** Soluble sugar content. **(B)** Proline content. **(C)** Soluble protein content. **(D)** Soluble protein expression. CK, control; A, ABA treatment under normal conditions; B, B2 treatment under normal conditions; S, salinity treatment; S + A, ABA treatment under salt stress; S + B, B2 treatment under salt stress. Data are presented as treatments mean with SE (*n* = 3). Bars with the same letter are not significantly different at *P* = 0.05.

Under salt stress, plants can reduce osmotic potential and adapt to the salt environment by synthesizing and accumulating osmotic regulatory substances. To explore the ability of wheat seedlings to resist salt stress, the contents of osmolytes were measured, including soluble sugar, proline, and soluble protein (Figure 4). Salinity increased the content of soluble sugar and soluble protein, while decreased the content of proline. Exogenous application of ABA and B2 had no significant effect on the content of soluble sugar and protein under neither condition (Figures 4A,C). However, exogenous applications decreased the proline content under both normal and salinity conditions compared with untreated control and salt treatment (Figure 4B). The expression of soluble protein was also measured, but the differences between the treatments were not significant under both normal and stress conditions (Figure 4D).

### Effects of exogenous B2 on content of endogenous hormones

**FIGURE 5:**
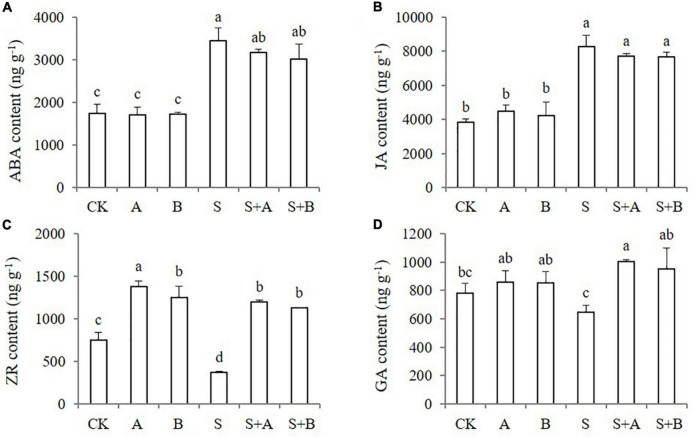
Effects of B2 on the endogenous hormone contents in leaves of wheat seedlings after salt treatments for 6 days. **(A)** ABA. **(B)** JA. **(C)** ZR. **(D)** GA. CK, control; A, ABA treatment under normal condition; B, B2 treatment under normal conditions; S, salinity treatment; S + A, ABA treatment under salt stress; S + B, B2 treatment under salt stress. Data are presented as treatment mean with SE (*n* = 3). Bars with the same letter are not significantly different at *P* = 0.05.

As known, plant hormones play an important role in stress response. In order to explore whether B2 is involved in regulating other plant hormones in the process of improving wheat tolerance to salt stress, the contents of endogenous hormones were measured, including ABA, JA, ZR, and GA (Figure 5). Under normal conditions, the contents of ABA, JA, and GA were not affected by the application of exogenous ABA and B2, while the contents of ZR were increased. Under salt-stressed conditions, the contents of ABA and JA were significantly induced by salinity stress, and the ZR content was obviously decreased. The exogenous applications of ABA and B2 significantly increased the contents of ZR and GA but had no significant effect on the content of ABA and JA.

### Effects of exogenous B2 on activities of antioxidant enzyme and 1,1-diphenyl-2-picrylhydrazyl scavenging

**FIGURE 6:**
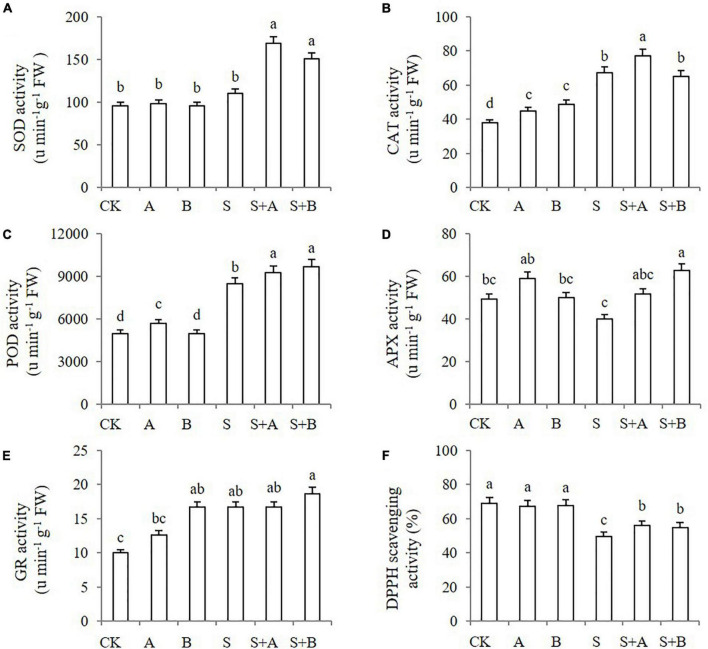
Effects of B2 on the activity of antioxidant enzymes and the 1,1-diphenyl-2-picrylhydrazyl (DPPH) scavenging in leaves of wheat seedlings after salt treatments for 6 days. **(A)** SOD. **(B)** CAT. **(C)** POD. **(D)** APX. **(E)** GR. **(F)** Activity of DPPH scavenging. CK, control; A, ABA treatment under normal conditions; B, B2 treatment under normal conditions; S, salinity treatment; S + A, ABA treatment under salt stress; S + B, B2 treatment under salt stress. Data are presented as treatments mean with SE (*n* = 3). Bars with the same letter are not significantly different at *P* = 0.05.

Under abiotic stresses, antioxidant enzymes have a role in ROS detoxification, which contributes to ROS scavenging. We measured the activities of antioxidant enzymes including SOD, CAT, POD, APX, and GR in wheat seedlings under different treatments for 6 days. ABA and B2 treatments worked differently on the enzymes (Figures 6A–E). Under normal conditions, ABA treatment increased the activity of CAT and POD, while B2 treatment increased the activity of CAT and GR. Under salinity conditions, the activities of SOD and APX were not affected but were significantly increased by the application of ABA and/or B2. Under salt stress, the effect of ABA on APX was not significant; however, the effect of B2 was significant (according to Figure 6D). The activity of CAT, POD, and GR was stimulated by salt stress. Under salt stress, ABA increased the activity of SOD, CAT, and POD, while B2 increased the activity of SOD, POD, and APX.

The non-enzymatic antioxidant activity was also estimated by monitoring DPPH scavenging (Figure 6F). Neither molecule affected the activity under unsalted conditions. Under salt stress, DPPH scavenging activity decreased significantly compared with the control. Both ABA and B2 treatment increased DPPH scavenging significantly, thus alleviating salt stress.

**FIGURE 7:**
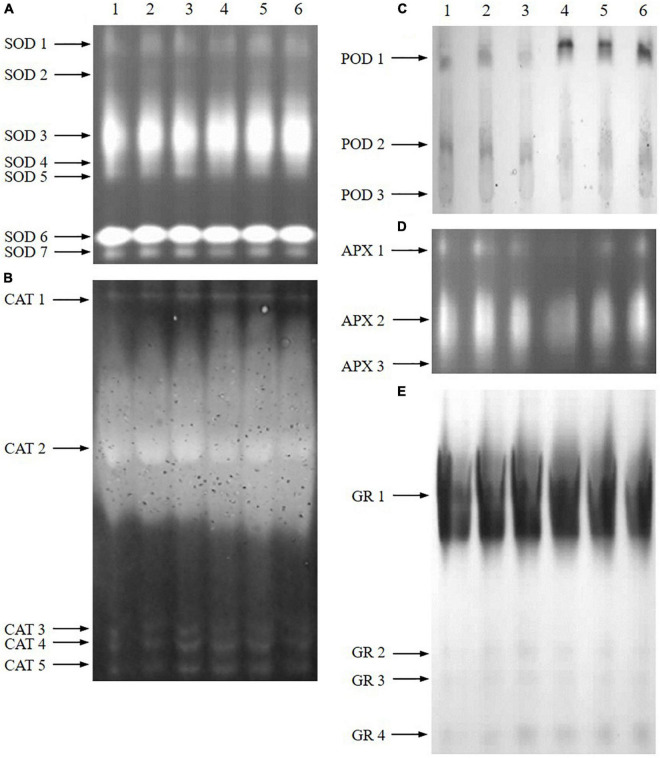
Native PAGE of antioxidant enzymes in leaves of wheat seedlings after salt treatments for 6 days. **(A)** SOD. **(B)** CAT. **(C)** POD. **(D)** APX. **(E)** GR (lane 1: control; lane 2: ABA; lane 3: B2; lane 4: salt; lane 5: salt + ABA; lane 6: salt + B2).

Native PAGE of antioxidant enzymes further revealed that the affection was not the same for all of the antioxidant enzyme isoforms (Figure 7). Under normal conditions, ABA treatment increased the intensity of CAT3/4/5, POD1, and GR2/3/4, while B2 treatment increased the intensity of CAT3/4/5 and GR2/3/4, which were consistent with the results of enzyme activity. The intensity of SOD1, SOD3, POD1, and GR4 increased under salt treatment, while APX2 and CAT2 decreased significantly. Pretreatment with ABA and B2 led to significant increases in SOD3 (particularly) and POD1/2 compared with salt-treated samples under salt treatment. Meanwhile, B2 also significantly increased the intensity of APX1/2, which is different from the results of enzyme activity.

### Effects of exogenous B2 on the expression of *TaSOS1* and *TaTIP2;2* genes

**FIGURE 8:**
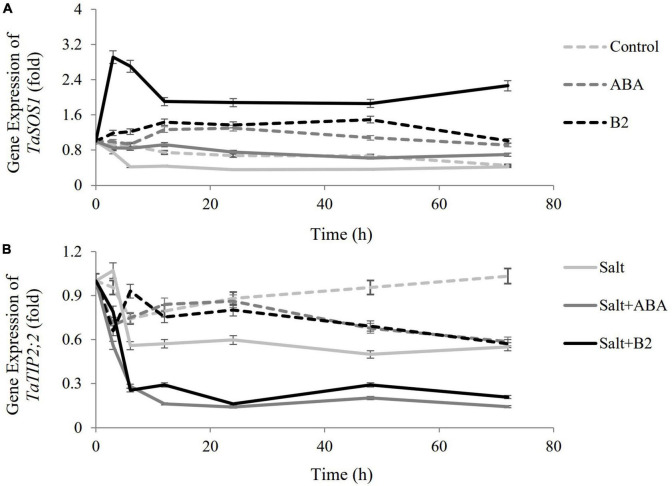
Effects of B2 on the expression of salt-responsive genes in wheat seedlings after salt treatments for 6 days. **(A)** Gene expression of *TaSOS1* (a transmembrane NA^+^/H^+^ antiporter). **(B)** Gene expression *TaSOS2*;*2* (a wheat aquaporin gene). Control; ABA, ABA treatment under normal conditions; B2, B2 treatment under normal conditions; salt, salinity treatment; salt + ABA, ABA treatment under salt stress; salt + B2, B2 treatment under salt stress. Data are presented as treatments mean with SE (*n* = 3). Bars with the same letter are not significantly different at *P* = 0.05.

In order to explore the regulation mechanisms of B2, the expressions of *TaSOS1* (a transmembrane Na^+^/H^+^ antiporter) and *TaTIP2;2* (a wheat aquaporin gene) genes were measured (Figure 8). The results showed that the expressions of *TaSOS1* genes were upregulated, and those of *TaTIP2;2* were suppressed by ABA and B2 treatments under normal conditions compared with the control. Both gene expressions were suppressed by salinity. Under salt-stressed conditions, the *TaSOS1* gene was upregulated by ABA and was more significantly upregulated by B2 compared with the salt treatment, while *TaTIP2;2* genes were suppressed.

## Discussion

Soil salinization is one of the major factors that negatively affect crop growth and limit agricultural production (Iqbal et al., 2018). Improving the salt tolerance of crops is essential for sustainable agriculture. To cope with such adverse conditions, plants develop certain internal mechanisms, but these mechanisms often fail to tolerate severe stresses. Among the various strategies, the application of plant growth regulators (PGRs) has gained significant attention to induce salt tolerance in plants (Rademacher, 2015). The application of ABA is an effective method to improving salt tolerance in plants (Iqbal et al., 2014). However, ABA is limited as a plant growth regulator due to its instable structure and being easily degraded in plant. In this study, we synthesized two ABA functional analogs and evaluated the function of alleviating the adverse effects of salinity in wheat seedlings. It was found that the effect of B2 was similar to ABA and better than B5 at 0.01 μM. Therefore, B2 was chosen as the target compound for further evaluation. Biomass yield, that is, dry weight, root/shoot ratio, and plant height, and physiological characteristics, that is, ROS generation and cell membrane integrity, osmolyte and endogenous hormone content, activities of antioxidant enzymes, DPPH scavenging, activity staining of antioxidase isozymes, and expression of *TaSOS1* and *TaTIP2* genes in wheat seedlings under normal or salinity conditions with different exogenous treatment or not were investigated in this study.

Salinity stresses could disturb normal growth of plants (Sharma et al., 2019). In this study, the results showed that the differences in the aforementioned parameters of biomass and physiological characteristics between untreated plants and B2-treated plants were not significant under normal conditions. Salt stress significantly affected most parameters investigated in this study. The applications of ABA and B2 could ameliorate salt-induced growth inhibition in wheat seedlings. Responses of wheat seedlings treated by ABA or B2 to salt treatment were partially different from each other, indicating that the physiological and biochemical mechanisms of B2 and ABA were different.

One of the initial effects of salinity stress on plants is the reduction of growth, which leads to the reduction of biomass. Salinity inhibits plant growth in two ways. One is the water-deficit or osmotic effect on the ability of plants to absorb water, which leads to slower growth. The other is the ion-excess or salt-specific effect on transpiration stream, which eventually injure cells in leaves and furtherly reduce plant growth (Parihar et al., 2015). The salinity in the root zone can inhibit root growth and increase leaf thickness. It has been shown that salt stress decreased the biomass of various plants in comparison with that of plants under normal conditions (Ahmad et al., 2019). In this study, salinity stress could significantly reduce the biomass of wheat seedlings, including dry weight, root/shoot ratio, and plant height, which was in agreement with previous studies. The exogenous B2 treatment could recover the amount of dry matter to a normal level. Plant development is also affected under abiotic stresses by various physiological and biochemical mechanisms, such as antioxidant systems and hormonal signaling (Oyiga et al., 2016).

Under salt stress, plants encounter three main challenges, including osmotic stress, ion toxicity, and oxidative damage. The generation and accumulation of ROS are usually induced by osmotic stress and ionic stress, which lead to oxidative damage (Parihar et al., 2015). Several research studies have revealed that the content of H_2_O_2_ and O_2_^–^ are significantly induced in different plants under salinity stress, such as cotton (Wang et al., 2016), Mung bean (Ahmad et al., 2019), *Brassica juncea* (Kaur et al., 2018), and *Lepidium draba* (Goharrizi et al., 2019). The results of the H_2_O_2_ content and generation rate of O_2_^–^ are similar to the previous results reported by Goharrizi et al. (2019). In our study, the H_2_O_2_ content and generation rate of O_2_^–^ in young wheat leaves were not affected by the applications of ABA under normal conditions in comparison with those of control plants, while the applications of B2 decreased the H_2_O_2_ content. As expected, the accumulation of both O_2_^–^ and H_2_O_2_ in salt-treated plants increased significantly, which were reduced by the exogenous ABA and B2 treatments. Overall, the exogenous applications could eliminate the impact of salinity on the generation rates of O_2_^–^ and H_2_O_2_ content in salt-stressed wheat seedlings, and B2 showed effects similar to ABA.

The accumulation of O_2_^–^ and H_2_O_2_ in plant cells leads to membrane damage and electrolyte leakage (Wang et al., 2012). The MDA content and electrolyte leakage are important indexes for plant resistance *via* lipid peroxidation and plasma membrane. Phytohormones could trigger the biosynthesis of osmolytes in plants growing under various environmental conditions (Sharma et al., 2019). Under stress conditions, ABA maintains the osmotic adjustments by regulating the biosynthesis and of accumulation of osmolytes in plant cells (Sarafraz-Ardakani et al., 2014; Karimi and Ershadi, 2015). In this study, the salt-treated plants exhibited increased accumulation of MDA and electrolyte leakage, which were similar to a previous research (Goharrizi et al., 2019). The MDA content was reduced by both exogenous ABA and B2 under normal and salt stress conditions. The electrolyte leakage content was decreased by exogenous ABA and B2 treatments only under salt stress conditions. Exogenous application of ABA and B2 was proved to relieve the membrane damage induced by salt stress.

Accumulations of carbohydrates, such as sugar and starch, are elevated under salt stress (Vijayalakshmi et al., 2016). It has been proved that plants increase the contents of sugars and other compatible solutes to decrease osmotic damage under salinity stress (Ashraf and Foolad, 2007). The major role of these carbohydrates involves carbon storage, osmo-protection, and scavenging of reactive oxygen species to relieve stress (Yancey, 1994). The sucrose content was found to increase in tomato (*Solanum lycopersicum*) under salinity by increasing the activity of sucrose phosphate synthase (Pressman et al., 2012). The results showed that the soluble protein content in *Astragalus membranaceus* var. *mongolicus* seedlings increased by the increase in the chloride concentration (Zhao et al., 2016). Salt stress induced the production of new proteins, meanwhile, increased the mass fraction of original proteins, thus causing an increase in the soluble protein content (Bertazzini et al., 2018).

To prevent oxidative damage induced by salt stress, plants have developed an antioxidant system, which comprises antioxidants and antioxidant enzymes, to maintain a dynamic balance of ROS. Enzymatic ROS scavenging mechanisms mainly include SOD, POD, CAT, APX, and GR (Islam et al., 2021). A previous study revealed that ABA induced the activities of POD and CAT under drought conditions (Sarafraz-Ardakani et al., 2014). When exogenous B2 was applied, the activities of SOD, POD, and APX were significantly increased compared with those with no exogenous application under salt stress. The exogenous application of B2 triggers the antioxidant enzyme systems of plants under salt stress and thus decreased the oxidative stress. Despite the activity of SOD, CAT, POD, APX, and GR form a network to scavenge ROS, plants also possess a non-enzymatic antioxidant. DPPH radical scavenging activity reflects the potential of non-enzymatic antioxidant activity. The high level of DPPH radical scavenging has been associated with increased salinity tolerance (Xie et al., 2007). In general, the antioxidant system was significantly stimulated by B2, similar to that by ABA, contributing to enhance performance under salt stress conditions at the whole plant level.

In conclusion, the exogenous ABA analog B2 showed effects similar to ABA and could alleviate the damage of salt stress to wheat seedlings. In terms of physiology, B2 reduced electrolyte leakage and lipid peroxidation and affected the hormone and antioxidant response under salt stress. While in terms of growth performance, the application of B2 promoted the growth of wheat seedlings under normal conditions, while increased the plant height and dry matter to a stress-free level under salinity conditions. Although the mechanism still needs to be further studied, the present results can support the conclusion that the exogenous application of B2 could be a feasible way to improve the salt tolerance of winter wheat seedlings. ABA plays an important role in plant growth and development, such as seed dormancy and adaptation to many abiotic stresses. Although B2 mimics the functions of ABA, further detections will be required to establish which responses of ABA are activated by B2. Before applying for agricultural purposes, it is necessary to perform toxicological assays. The value of plant growth regulators is that they can be applied on demand, whereas crop genetics cannot be implemented in one growing season. A common role of genetics and chemistry is likely to have maximum benefit (Cao et al., 2017). B2 is a new chemical tool for improving plant salinity tolerance.

## Data availability statement

The original contributions presented in this study are included in the article/Supplementary material, further inquiries can be directed to the corresponding author.

## Author contributions

CY and LD designed the study. CY, FZ, and ZR performed the experiments. WT, LJ, SC, ZX, YX, and YZ analyzed the data. CY, RW, and LD wrote the manuscript. All authors contributed to the article and approved the submitted version.
