# Supplementary material for: B2, an abscisic acid mimic, improves salinity tolerance in winter wheat seedlings via improving activity of antioxidant enzymes
Source: Front Plant Sci. 2022 Sep 27;13:916287. doi: 10.3389/fpls.2022.916287 (PMC9551657; doi:10.3389/fpls.2022.916287)
Supplement: Supplementary file 1 [file Data_Sheet_1.docx]

**Supplement Materials**

**Table S1** The potency of different analogues on wheat seed germination

| Treatments | Germination rate(%) | |
| --- | --- | --- |
|  | 1000 µmol/L | 5000 µmol/L |
| CK | 93.3 | 93.3 |
| ABA | 94.7 | 56.0 |
| **B2** | 92.0 | 40.0 |
| **B5** | 97.3 | 46.7 |

**Table S2** Primers used for RT-qPCR verification

| Name | Sequence |
| --- | --- |
| *TaSOS1-*F | 5’- cttcgcgctcatagaattgc -3’ |
| *TaSOS1-*R | 5’- taatgcacgaagcacctgag -3’ |
| *TaTIP2;2-*F | 5’- actccttcagcttggcctct -3’ |
| *TaTIP2;2-*R | 5’- atatcgccacggcaatcag -3’ |
| *β-actin*-F | 5’- acgtggcccttgattatgag -3’ |
| *β-actin*-R | 5’- caacatgagatggctggaac -3’ |
